# Privacy-Aware Architectures for NFC and RFID Sensors in Healthcare Applications

**DOI:** 10.3390/s22249692

**Published:** 2022-12-10

**Authors:** Emanuele Raso, Giulio Maria Bianco, Lorenzo Bracciale, Gaetano Marrocco, Cecilia Occhiuzzi, Pierpaolo Loreti

**Affiliations:** 1 Department of Electronic Engineering, University of Rome Tor Vergata, 00133 Roma, Italy; 2 Pervasive Electromagnetics Lab, Department of Civil Engineering and Computer Science Engineering, University of Rome Tor Vergata, 00133 Roma, Italy

**Keywords:** attribute-based encryption, body-area Internet of Things, cybersecurity, healthcare Internet of Things, Near Field Communication, point-of-care, proxy re-encryption, radio frequency identification

## Abstract

World population and life expectancy have increased steadily in recent years, raising issues regarding access to medical treatments and related expenses. Through last-generation medical sensors, NFC (Near Field Communication) and radio frequency identification (RFID) technologies can enable healthcare internet of things (H-IoT) systems to improve the quality of care while reducing costs. Moreover, the adoption of point-of-care (PoC) testing, performed whenever care is needed to return prompt feedback to the patient, can generate great synergy with NFC/RFID H-IoT systems. However, medical data are extremely sensitive and require careful management and storage to protect patients from malicious actors, so secure system architectures must be conceived for real scenarios. Existing studies do not analyze the security of raw data from the radiofrequency link to cloud-based sharing. Therefore, two novel cloud-based system architectures for data collected from NFC/RFID medical sensors are proposed in this paper. Privacy during data collection is ensured using a set of classical countermeasures selected based on the scientific literature. Then, data can be shared with the medical team using one of two architectures: in the first one, the medical system manages all data accesses, whereas in the second one, the patient defines the access policies. Comprehensive analysis of the H-IoT system can be useful for fostering research on the security of wearable wireless sensors. Moreover, the proposed architectures can be implemented for deploying and testing NFC/RFID-based healthcare applications, such as, for instance, domestic PoCs.

## 1. Introduction

**Figure 1:**
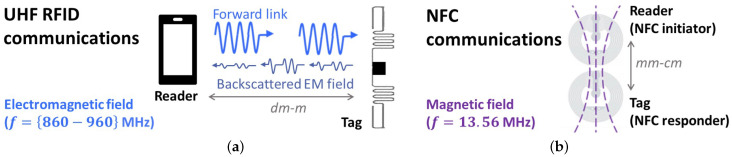
Scheme of the typical communications through RFID devices: electromagnetic links of (**a**) UHF RFID, with the operation frequencies considered in this work, and (**b**) NFC.

Thanks to steady advancements in medicine, life expectancy has risen worldwide since 1990, but the disability burden, mainly caused by chronic illnesses, has increased too [1], resulting in high costs for healthcare systems and states [2]. The cost-effectiveness of medical treatments can improve enormously through digital medicine, such as deep learning neural networks for diagnosing diseases [3] or remote patient monitoring [4], hence addressing the challenges of chronic medical conditions that are more and more common. Radiofrequency identification (RFID) technology can significantly help to face such challenges. At its core, an RFID system is constituted by a reader device, which can interrogate the surroundings in a wireless mode to receive a response from nearby tags; however, features such as the maximum reading distance and anti-collision management depend on the specifically implemented system [5]. In further detail, the NFC (Near Field Communication) set of protocols is an evolution of the RFID, which communicates in a peer-to-peer way at the specific frequency of 13.56 MHz [6], exploiting magnetic inductive coupling, whereas ultra-high frequency (UHF) RFID devices usually communicate in regional, unlicensed sub-bands of the 860–960 MHz band through electromagnetic backscattering. Figure 1 summarizes the main differences between the two RFID technologies considered hereafter, including their standard reading distances.

In recent years, NFC and UHF RFID have therefore enabled a myriad of healthcare Internet of Things (H-IoT) systems [7], including telemonitoring [8,9,10], assistive devices for sensorial-impaired patients [11,12], and also intra-body communications [13,14]. Among the main advantages of the two technologies is the low cost of the single tags, which allows them to be disposable and vastly deployed, especially in the case of battery-less ones. Thus, RFID sensors can become crucial for the delivery of personalised and precision treatments in the near future, allowing for the creation of points of care (PoCs) that can perform sensing and analysis while avoiding carrying samples to any lab [15], eventually combining the NFC and UHF RFID technologies to communicate the data to the doctor and maximising their respective strengths [16].

To enable continuous monitoring, NFC/RFID medical PoCs usually generate a huge amount of sensitive data that must be stored and shared with doctors. The security and privacy of this data must always be guaranteed. In such systems, much attention has been paid to the security of the radio frequency link, i.e., the signal from the sensors to the reader [17,18]. For instance, a lightweight mutual authentication protocol for tags and readers was proposed in [19] to ensure forward secrecy; however, the protocol needs additional complexity to withstand desynchronization attacks. Resistance to desynchronization can be achieved by mutual authentication through a pseudo-number generator and the messages’ timestamps [20]. Moreover, security models were developed and proposed to assess the protocols’ security, although multiple models should be used to analyze the robustness of a given protocol comprehensively [21]. On the contrary, secure information sharing with healthcare facilities or staff has received much less attention, and patients are often forced to send their sensitive data by e-mail or via instant messaging systems such as WhatsApp [22]. Although these systems allow the contingent problem to be solved, when the amount of data becomes large, it is necessary to resort to different solutions that properly use general-purpose cloud services.

Cloud-based architectures are convenient for managing the vast amount of data that can be generated by wireless sensors without overwhelming local computer systems [23,24]. Still, the use of external public services raises a two-level issue regarding the privacy of the patient: (*i*) The monitoring platform must comply with the regulatory framework on sensitive data, e.g., the European GDPR [25], and (*ii*) technical solutions must be adopted to ensure secure access to the data without involving the cloud service, which, in general, could be an *honest-but-curious* or even malevolent actor. Hence, ensuring privacy and data security in cloud services is a major issue, which has been widely discussed in the literature [26]. Some solutions based on classical PKI (public key infrastructure) technologies [27] have been proposed. However, to provide more flexible key management and access control, systems making use of modern cryptographic techniques such as homomorphic encryption (HE) [28] and functional encryption [29,30] have been presented.

### 1.1. Novelty over Previous Works

Existing literature in the RFID and NFC security is largely application-independent [31,32,33,34,35,36] and does not consider the peculiarities of the healthcare domain. Thus, published papers either detail specific attacks [37,38,39] or develop advanced defenses [40,41,42]. Particular care was devoted to authentication protocols [19,20,43,44,45,46], since they are the basis of the most crucial countermeasures. Previous research undoubtedly had the merit of addressing many of the possible attacks using a plethora of different techniques, whose efficacy was carefully evaluated.

Furthermore, regarding the transmission of the data from the reader to the doctor, many investigations have studied the secure sharing of electronic health records (EHRs) through standards such as HL7 [47]. Moreover, the exploitation of cryptographic techniques such as ABE has already been proposed by several researchers [48,49,50,51]. In such research, the health records are managed by the healthcare system and/or the medical staff. Instead, in the secure architecture here analyzed, raw sensor data gathered by the NFC and RFID readers has to be transmitted to the doctor, who will integrate them in the EHR only successively.

Overall, after a thorough search through the literature (see next Section 2 for more information on existing literature relevant to the present work), no previous research has investigated secure architectures for NFC and RFID medical sensors while both considering the possible vulnerabilities of wireless, electromagnetic links and the usage of a cloud provider other than the medical system. Indeed, the investigation of similar secure architectures has been gaining attention in recent years [16,48,52,53]. To the best of our knowledge, this is the first work on the security of raw medical data collected by sensors and then shared with the physician through such an NFC/RFID system.

### 1.2. Contribution and Structure of the Paper

In this article, we address the problems related to the security of the medical data collection and sharing. In particular, we analyse the collection of data through NFC/RFID in a domestic PoC representing a typical healthcare application enabled by radiofrequency identification and subsequent cloud-assisted data sharing. The literature on attacks against the tag-reader link is reviewed, and a set of known countermeasures to preserve the privacy of communications is conceived and shown. Afterwards, two secure cloud-based data-sharing architectures are presented, which make use of the advanced cryptographic techniques to provide a robust, flexible and fine-grained access control independently of the underlying cloud service. In the first architecture, relying on the ElGamal-based [54] HE and proxy re-encryption (PRE), the medical system (hereafter assumed to be a hospital for the sake of simplicity) is responsible for the management of both data access and cryptographic keys. In the second solution, based on the attribute-based encryption (ABE), the patient defines the access policies, while the medical system is responsible for the management of the cryptographic keys. Both the proposed architectures can use honest-but-curious cloud providers, so they do not have to be *fully trusted*, because they only see encrypted data. Consequently, the main contribution of this work is two-fold:

1. The security of NFC/RFID tag-reader links for medical data collection in a healthcare application is analyzed. Main threats are identified, and countermeasures are proposed based on the literature;
2. Two novel security architectures for cloud-based file storage/sharing (FSS) that make the cloud provider an *oblivious transfer* of files from patients to doctors are described.

The rest of the paper is structured as follows. Section 2 reviews the literature on the security of NFC and RFID in healthcare, focusing on the most relevant attacks for PoCs, and on the security of cloud-sharing architectures. Section 3 describes the system model, presenting the scenario and introducing the background on the system’s components: the NFC and RFID medical sensors, HE, PRE and ABE. Then, the security model is introduced in Section 4. In particular, a set of techniques for securing clear communications between the NFC/RFID sensor and the reader is proposed in Section 4.2, whereas the two data-sharing architectures are detailed in Section 4.3. Section 5 evaluates the computational burden due to the reader-doctor encrypted communications and, lastly, Section 6 concludes the work.

## 2. Related Work

### 2.1. Aim and Methodology of the Literature Review

A literature review highlights the recent trends and main threats in the security of radiofrequency identification to be considered for the deployment of PoCs. The trend analysis to draw the research lines was performed through the ScientoPy scientometric software [55], scouting both the SCOPUS and Web of Science (WoS) databases for reports without any temporal restriction.

As for the methodology, after the database searches, the first screening omitted the documents that were not journal articles, conference papers, reviews, proceeding papers, or articles in press. The second screening omitted duplicates, and then the relevant searches were merged to create a list of unique entries. The results were analyzed to evaluate the topic’s growth and trends. Finally, a set of recent related works were selected based on the abstract and the conclusions for rigorous study.

### 2.2. Security of NFC and RFID for Healthcare Applications

Since RFID is a pervasive technology that is commonly widespread, many reviews comprehensively investigate the topic of its security for generic applications, as, for instance [31,32,33]. However, H-IoT systems based on radiofrequency identification have gained momentum for innumerable uses, so specific security and privacy concerns that are still unaddressed have been raised [56].

**Figure 2:**
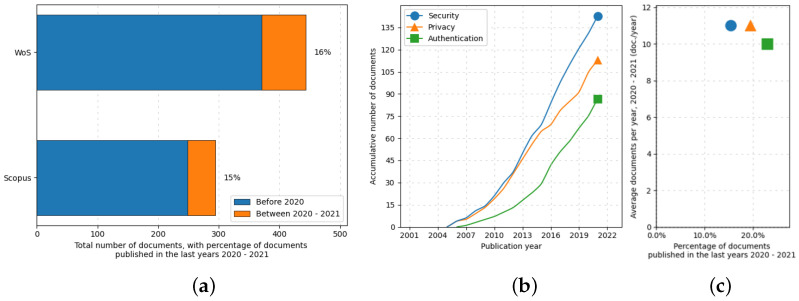
ScientoPy processing of the literature on NFC and RFID security in healthcare applications: (**a**) percentage of the works published since 2020 over all the relative literature, (**b**) documents vs. publication years based on keywords, and (**c**) average documents per year vs. the percentage of published documents between 2020 and 2021.

The search strings employed in the literature review and the number of reports after the subsequent screenings are summarized in Table 1. About 15% of the 764 unique reports identified were published since 2020, denoting an increased interest in the security of NFC/RFID medical devices from the scientific community beyond the sole authentication issue (Figure 2). Based on the rigorous study of the selected literature, the most common attacks to be considered in the tag-reader link are basically of the five types, discussed as follows: (*i*) skimming, (*ii*) eavesdropping, (*iii*) man-in-the-middle, (*iv*) power analysis attacks, and (*v*) timing attacks. It is worth noticing that, although typical reading distances of NFC are just a few centimeters, it is theoretically possible to attack the medical NFC devices nonetheless using sensitive hardware [37], and the attack range can be further extended by exploiting higher-order harmonics [38].

#### 2.2.1. Skimming

Wireless skimming happens when an RFID or NFC tag is secretly interrogated by a malicious reader and is performed by fraudsters to steal small sums of money [57]. This kind of attack must not be confused with the well-known mag-stripe skimming used by thieves to duplicate payment cards. In telemonitoring applications, wireless skimming can be exploited to obtain medical data when the wearer is unaware from distances much longer than the usual operating range. For instance, high-power readers can theoretically activate NFC tags up to 50 cm [38]. Even when access passwords protect the data, skimming the tag’s identifier can allow an adversary to clone the tag [39] so as to modify the medical history of the patient or compromise the system functionality. Hence, unauthorized interrogations should be prevented altogether.

#### 2.2.2. Eavesdropping

Eavesdropping occurs when the attacker intercepts transmitted data. Also known as *sniffing* or *snooping*, it is the most dangerous attack for RFID-based telemonitoring systems. Given the low cost and low memory of RFID tags, the physical-based defence is extremely convenient: as an example, reference [43] proposes the use of physical unclonable functions (PUF), namely, a set of delay circuits that can generate a unique random binary sequence based on the manufacturing of every single microchip. Although attackers can predict the password of the response if they have a sufficient number of PUF-response pairs, a low-cost single-use RFID medical sensor (such as [58]), could ensure the security of all transmitted data through PUF.

Another possible attack is active eavesdropping, whereby the eavesdropper transmits a continuous wave outside the FHSS (frequency-hopping spread spectrum) channels. Incapable of distinguishing between the attacker and the legitimate reader, the RFID tag will backscatter both signals, providing the attacker with more pieces of information [59]. A way to counter this more sophisticated attack is the use of artificial noise, possibly by a channel-conscious reader [34].

#### 2.2.3. Man-in-the-Middle

In man-in-the-middle (MIM) attacks, the attacker intercepts and possibly modifies the data between two legitimate parties before re-transmitting them to the intended recipient. These attacks are extremely difficult to perform due to the close communication range between reader and tag, and the peculiarities of the inductive and backscattering links. It is practically impossible to attack passive tags in this way [35]. However, since NFC/RFID sensors are resource-constrained devices due to the low cost per tag unit, the employed lightweight protocol could be vulnerable to specific attacks such as the HB (Hopper and Blum [60]) family of protocols vulnerable to the Gilbert-Robshaw-Sibert (GRS) attack [61]. Therefore, if active tags are employed for sensing the patient’s parameters, MIM attacks should be seriously contemplated when assessing the threat model, as in [62].

#### 2.2.4. Power Analysis Attacks

Among the side-channel attacks (SCA), simple (SPA) and differential power analyses (DPA) are the most relevant to telemonitoring security. SPA allows for deducing the 0 and 1 bits transmitted by observing the electromagnetic field perturbed by the tag. In the MIM case, the SPA is practically impossible to perform if the communication distance between the reader and the tag is a few centimeters, whereas it is a possible attack if a reading range of a few meters is exploited. In any case, some elliptic curve cryptography (ECC) protocols can protect the transmitted data [44]. Specifically, Liao’s ECC protocol [40] exploits the Montgomery ladder as an effective countermeasure to SPA [63].

The vulnerability to DPA can also be guarded by increasing the internal complexity of RFID integrated circuits (ICs) at the cost of the increased price per tag [36]. Examples of this approach are the adiabatic ICs that show uniform energy consumption during the operations [41]. Notably, adiabatic logic is highly efficient at the NFC frequency, whereas it is more challenging to use effectively at UHF frequencies [42].

#### 2.2.5. Timing Attacks

Timing attacks are performed by observing the time required to perform actions. For instance, it is possible for an attacker to understand which tag has been authenticated by simply measuring the time the reader needs to authenticate that specific tag, since this time is equal for each authentication [45]. As the time required to execute the steps prescribed by the NFC/RFID protocols depends on the state or responses of the tags, an attacker can recognize the tags based on timing in order to trace them [46]. Depending on the actual algorithm employed, timing attacks can even be exploited to recognize the transmitted key and decrypt messages [64].

### 2.3. Security of Cloud Sharing Architectures

#### 2.3.1. Public Cloud Security and Privacy

**Figure 3:**
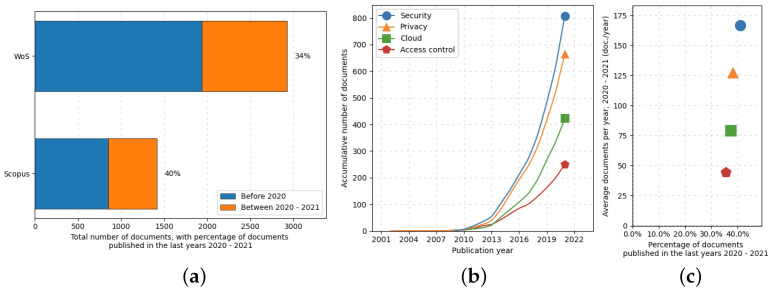
ScientoPy processing of the literature on privacy/security and access control in cloud applications: (**a**) percentage of works published since 2020 over all the relative literature, (**b**) number of published documents vs. publication years based on keywords, and (**c**) the average number of documents per year vs. percentage of published documents between 2020 and 2021.

Cloud-based services play a very significant role in several applications, given that they are fundamental building blocks of the whole Internet ecosystem. Outsourcing several IT facilities, such as e-mail servers or data storage, allows companies to focus on their business [65]. However, several issues related to the security/privacy of the data transferred to the cloud providers have been raised. In critical scenarios, e.g., healthcare (where sensitive data has to be processed), these concerns hinder the adoption of these kinds of services (particularly the ones based on public cloud systems). The need for solutions to these issues is also clearly visible in the literature. In fact, as shown in Figure 3, as interest in the cloud grows, so does the volume of work related to its security, privacy and access control. Exhaustive analyses of state-of-the-art technologies have been performed in [66,67,68]. Several solutions that make use of different technologies have been proposed for the healthcare scenario. In [69], authors propose a solution based on anonymisation and smart contracts to secure the transactions generated by mobile IoT devices. Regarding the management of access control, different solutions have been proposed, too. Some works [48,49,50] make use of advanced cryptographic schemes such as ABE and PRE and assess their security according to the random oracle model [70,71,72].

The search strings employed in the literature review and the number of reports after the subsequent screenings are summarized in Table 1. More than one-third of the 4936 unique reports identified have been published since 2020, denoting a significantly increased interest from the scientific community in the security of cloud systems for healthcare applications (see Figure 3).

#### 2.3.2. Advanced Healthcare Data Sharing

Many works related to medical data sharing deal with the problem of sharing Electronic Health Records (EHRs) between healthcare infrastructures, healthcare-specific clouds, patient devices, etc. In this context, the main role is played by the HL7 family of standards, which offers advanced options with FHIR technology [47].

However, several works propose solutions based on advanced cryptographic techniques such as ABE to solve specific privacy and security issues that arise when cloud services are used, while limiting the complexity of the cryptographic key management and sharing systems. For example, in [49], the authors consider the scenario of using generic cloud services, to which the patient delegates the management of his/her medical data. The authors try to solve the problem of the typical loss of access control. Therefore, the work proposes integrating the ABE cryptographic technique into EHRs to allow the user to define access policies to their information. The application of ABE has been observed over the years to entail numerous implementation issues, particularly considering the complexity of the healthcare world. For this reason, a system based on Multi-Authority ABE (MA-ABE) is presented in [51]. This makes key management even simpler, allowing a scalable solution to be realised, at least from this point of view. However, the use of the MA-ABE version has not yet been standardised by the relevant bodies, and its implementation is proprietary and not widely applied in an industrial environment.

Reference [48] increases the level of complexity and protection offered to address the problem of data sharing between multiple hospitals, and proposes an architecture that uses ABE as a cryptographic technique, integrating it with Secret Sharing techniques to improve the privacy of the infrastructure. Such a system is extremely complex and requires the creation of a single infrastructure for the entire healthcare system, which in many real-life scenarios does not seem practically feasible.

Finally, it is worth mentioning several works that study the integration of cryptographic techniques in the blockchain to implement information-sharing infrastructures that respect patient privacy regulations [73]. Indeed, the integration of privacy-enhancing techniques with blockchain has already proven its effectiveness in other fields for defining secure distributed infrastructures [74].

## 3. Scenario, System Model and Components

### 3.1. Scenario and System Model

**Figure 4:**
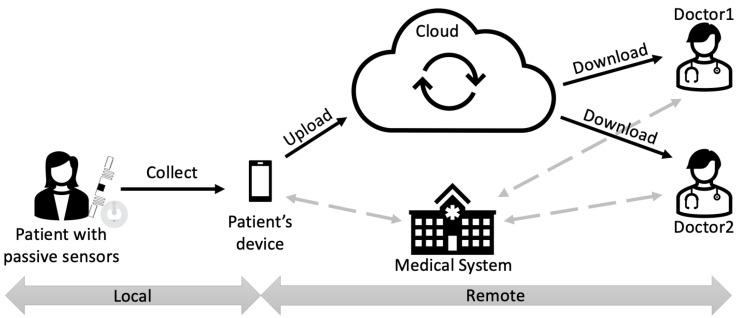
Considered scenario of NFC and RFID sensors for a healthcare application.

Let us consider the scenario in Figure 4 with a domestic PoC, where we suppose a (female) patient uses her own smartphone or laptop to store data on her own health condition collected from some NFC/RFID medical sensors. At the end of data collection, she wants to share all this information with her doctor in a *privacy-preserving* way. Furthermore, it would be desirable that these data could also be accessible to other healthcare entities who might request them, for example, in case of an emergency. Since the amount of data for each user could grow really quickly, and the communication between the patient and the doctor is most likely asynchronous, the use of a sharing service with underlying storage is a good choice in order to provide enough storage, as well as to decouple and desynchronise the two parties. Moreover, to avoid the costs of implementing and operating an on-premises service, using a public cloud-based file-sharing service is definitely the best solution.

As shown in Figure 4, the system can be divided into two parts, a local one and a remote one. All operations related to collecting data from medical sensors and saving data take place within the local part; the remote part is related to secure cloud-based data sharing. Due to the low-range characteristic of the local part, the remote one raises the most privacy concerns related to this scenario.

According to this discussion, we consider four actors:

1. The *Cloud Provider*, one of the existing commercial providers which offer file storage, sharing and synchronisation services (e.g., Dropbox, Google Drive, ownCloud, etc.);
2. The *patient(s)*, who stores on her smartphone or laptop the data collected from the medical sensors and has to be able to share it with the medical staff;
3. The *Medical Personnel (or Staff)*, whose members have to be able to download and access data shared by the patients;
4. The *Medical System*, the entity responsible for the management of the authorisations of the Medical Personnel to access patients’ data.

The aforementioned actors and their interactions are depicted in Figure 4. Patients and Medical Personnel use the Cloud Provider to share protected data, while they interact with the Medical System externally to obtain the cryptographic keys independently.

### 3.2. Types and Protocols of NFC and RFID Medical Sensors

**Figure 5:**
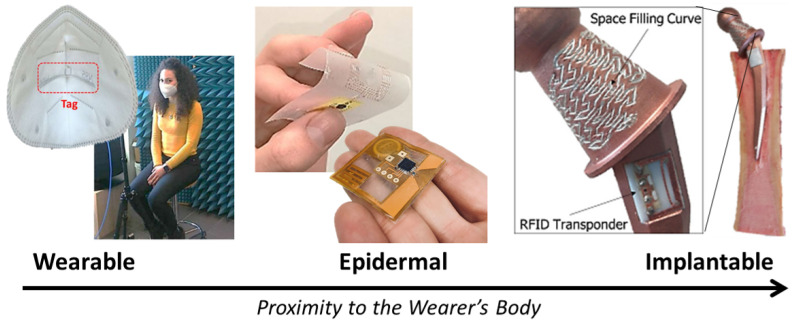
Examples of NFC and UHF RFID medical sensors that are categorized into three classes: wearable, epidermal, and implantable. From left to right: a wearable UHF RFID temperature sensor for monitoring coughing (image adapted with permission from Ref. [77]. © 2021, IEEE); NFC (image adapted with permission from Ref. [76]. © 2022, IEEE) and RFID (image adapted with permission from Ref. [78]. © 2022, Elsevier) sensor for analysing sweat; an implantable RFID sensor for detecting cracks (image adapted with permission from Ref. [13]. © 2021, IEEE).

The most widespread and important NFC and UHF-RFID medical sensors are the body-worn tags for sensing biosignals [7]. They can be broadly categorized into two classes: *wearable* tags, usually embedded in pieces of clothing, and *epidermal* tags, which are extremely thin devices similar to patches and mostly imperceptible for the wearer [75]. Due to the losses of biological tissue, epidermal UHF RFID sensors typically reach shorter reading distances than wearable ones [75], whereas NFC sensors maintain roughly the same level of performance thanks to their high-frequency (HF) working frequency, which is less affected by the human body’s presence [76]. An additional third category of medical sensors, the *implantable* ones, are characterized by even shorter communication ranges and are still a topic under research  [13,14]; therefore, we will hereafter focus on wearable and epidermal sensors. Such tags can sense physical or chemical measurements, such as temperature and pH [77,78]. Examples of NFC/RFID sensors for healthcare applications are illustrated in Figure 5.

Moreover, the security of the various existing RFID protocols varies [79]. This work regards the threats most relevant to the security of telemonitoring systems, using two protocols commonly employed for medical sensing: GS1’s EPC Gen2 (UHF RFID) and ISO/IEC 14443 (NFC). The only security tool in the UHF EPC Gen 2 is a 16-bit random number generator (RNG) [80] **and the ISO/IEC 14443 does not implement any encryption** by itself [81].

### 3.3. Homomorphic Encryption and Proxy Re-Encryption

Homomorphic encryption is a cryptographic technique that allows users to perform computations on the encrypted data (*ciphertext*) without first decrypting it. The results of these computations are also in encrypted form, and, once decrypted, the users observe results identical to the ones produced from the direct application on these computations on unencrypted data (*plaintext*). The problem of *secure computation* was introduced in 1978 [82], less than a year after the release of RSA (Rivest-Shamir-Adelman). It remained an open problem for more than 30 years, during which several partial homomorphic encryption (PHE) schemes were proposed, e.g., ElGamal [83] and Paillier [84] cryptosystems. The very first solution of a fully homomorphic encryption (FHE) scheme was proposed in 2009 [85]. In the following years, second-, third-, and also fourth-generation FHE schemes have been proposed [86,87,88,89,90]. Thanks to its properties, HE introduced the concept of privacy-preserving operations, and it has found a large variety of applications [91], e.g., storage, also in critical scenarios such as healthcare [92,93,94]. It is also used to provide role-based access control (RBAC) [95,96]. Moreover, it is the building block of secure multi-party computation (SMPC), which allows different parties to jointly compute a function over their input data, while keeping them private.

Re-encryption is a cryptographic technique that allows the transformation of a ciphertext, which has been encrypted initially for a specific user, into a new ciphertext so that it can be decrypted by another user. In classical solutions, firstly, the ciphertext is decrypted using the cryptographic key related to the old user, and then the resulting plaintext is encrypted using the key related to the new user. Since during the process, data are decrypted, a leakage of its content is possible, especially if this process is not directly performed by the data owner. If the data contain sensitive information, this operation could be critical and, therefore, should be left on the shoulders of the data owner. Proxy re-encryption is a cryptographic technique that allows a third party (*proxy*) to perform the re-encryption operation. In contrast with classical solutions, the most important property of PRE is that it does not require the decryption of the data, but the operation is directly executed on the original ciphertext. Thus, during the process, there is no leakage of the data content, thus preserving its privacy. Moreover, the old decryption key is not compromised and can be used in the future. Therefore, also in critical scenarios (e.g., healthcare, government), the data owner can delegate another entity to perform these operations without concerns about the leakage of sensitive information. PRE has been extensively studied in the literature due to the underlying characteristics related to trying to provide a transformation function that is unidirectional and transitive (actually, the only one known uses HE). PRE was first proposed by Blaze, Bleumer, and Strauss [97] in 1998. Their scheme is based on ElGamal on prime-order groups. In 2006, Ateniese et al. [98] proposed a scheme based on bilinear pairings. More advanced solutions were presented afterwards. For instance, in [72], the authors propose an identity-based proxy re-encryption scheme, while a lattice-based scheme is presented in [99].

### 3.4. Attribute-Based Encryption

Attribute-based encryption is a relatively recent asymmetric encryption technique originally conceived by Amit Sahai and Brent Waters in 2005 [100], then further developed in 2006 [101], while in 2007, the ciphertext-policy attribute-based encryption (CP-ABE) was presented as a new method for implementing complex access control on encrypted data [71]. The CP-ABE scheme, the most widely used ABE configuration, works as follows: a plaintext is encrypted with an ABE public key together with an access policy, which is a set of attributes combined with logical constructs such as AND, OR or threshold gates (k-out-of-n). Attributes are labels (i.e., strings) whose semantics may be adapted case by case: for instance, attributes can be CARDIOLOGIST, SURGEON or NEUROLOGIST and a policy applied to an encrypted message can be “CARDIOLOGIST” OR “SURGEON”. Each user is provided with a secret key, released by an entity called *Attribute Authority*, which is associated with a set of attributes. Only the user with attributes satisfying the policy can decrypt the message. Using ABE in the proposed scenario is particularly interesting since the true recipients of a message can be unknown at the time of encryption (just the policy is needed), so granting a new user access to a data repository does not require any change on the ciphertext. How to revoke some attributes to a user after they have been granted is a major open issue. The most adopted solutions with ABE use time-varying attributes, which implement versioning. For example, hospital surgeons could periodically receive their keys with attributes such as SURGEON_V1 and, one week later, SURGEON_V2. Preventing data access to a surgeon can be enforced simply by stopping providing her with the new keys with the new version of the attributes. This strategy implies that, in order to prevent long policies, the whole data repository must be re-encrypted with the policy containing the new attributes, which is expensive from a bandwidth and time point of view.

## 4. Security Architecture

### 4.1. Trust Model

We want to protect patients’ sensitive data against any possible unauthorised access attempts. Thus, we want to provide protection against other patients who could use the very same cloud storage service, as well as medical personnel for whom the data are non-destined. We also want to prevent the Cloud Provider from accessing users’ data.

We assume the medical system ca be trusted because it is responsible for providing patients and medical personnel with cryptographic keys and access authorisations. On the other hand, we assume an honest-but-curious trust model for the Cloud Provider, which is not trusted to read the data but can be trusted to perform any requested operation. We also assume honest-but-curious users (and revoked users) who may attempt to access data to which they are not (or no longer) authorised. Thus, this trust model is the motivation behind why we store data in an encrypted form and do not want the Cloud Provider to manage the access keys for the users.

### 4.2. Secure Tag-Reader Link

**Figure 6:**
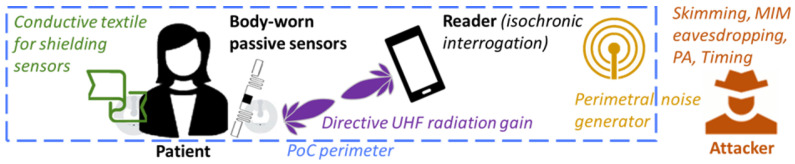
Sketch representing a secured tag-reader link in a domestic PoC.

Concerning the first part of the system model, based on the main threats recalled in Section 2.2, the tag-reader link can be secured by combining a set of simple countermeasures. Firstly, to prevent MIM attacks and strongly reduce the risk of skimming and eavesdropping, active tags should be avoided and only passive or battery-assistive-passive (BAP; i.e., tags wherein a battery is used to feed internal circuitry but is not exploited to start nor foster communications) transceivers should be used for sensing. In this way, the range of the attacker is lowered to a few meters [37]. Then, the epidermal/wearable sensors should be shielded when they are not being used to avoid skimming; for instance, a data-logging UHF RFID board [102] for monitoring outdoor activity should follow a challenge-response procedure not to respond to any unexpected query, and an epidermal/wearable NFC sensor [76] should be shielded with a conductive textile to avoid unauthorized accesses in crowded areas. Timing attacks can be prevented through isochronic code, which employs delays or fixed timings for each operation performed by the reader without hindering the sensing of biosignals that are slow-varying signals. Additional countermeasures can be adopted against eavesdropping to further secure the tag-reader link: directive UHF RFID antennas make it more difficult eavesdropping, and simple noise generators installed on the perimeter of the PoC can completely shadow backscattering communications from a distance. The noise generators should also shadow the strongest higher-order harmonics. Figure 6 depicts an example of a secured sensor-reader link for healthcare applications; naturally, based on the sensitivity of the actual data to be transmitted, a subset of these precautions can be deemed sufficient by the system designer.

The countermeasures detailed above can secure even unencrypted tag-reader links in PoC scenarios typical of COTS EPC Gen 2 and ISO/IEC 14443 devices. However, it is worth noting that encrypted tag-reader communications are currently surging. For instance, the AES-128 bit encryption is supported by the MIFARE NFC protocol [103] and the NXP^®^ UCODE^®^ DNA Track integrated circuit [104] can even provide tag-specific keys, which are extremely effective if low-cost single-use medical tags are deployed. The NFC/RFID-enabled healthcare applications can greatly benefit from this last-generation hardware, further securing the tag-reader link.

### 4.3. Secure Sharing

We propose two different secure-sharing strategies: both of them provide *data-centric security*, so they do not rely on the robustness of the underlying infrastructure but on the strength of the adopted cryptographic schemes. Both solutions are managed by the Medical System, which is responsible for the generation and distribution of the cryptographic keys used to encrypt/decrypt data. Since the cloud-based storage/sharing service stores only encrypted data, it is an oblivious data transfer service.

The two solutions differ from a cryptographic point of view according to the properties deriving from the two different adopted schemes. On the contrary, they are based on almost identical interactions and are comprised of the following four components:

1. a *Key Management Service* (KMS), run by the medical system, responsible for the generation and distribution to patients and medical personnel members of the cryptographic keys for the encryption and decryption operations;
2. A public *File Storage/Sharing* service, offered by the honest-but-curious Cloud Service Provider (e.g., Dropbox), used as an oblivious transfer to store and share the protected data that is shared between patients and medical personnel;
3. The *Data Owner* (DO), i.e., a patient who collects sensitive data from the medical sensors of the domestic PoC and transfers it from a smartphone/laptop to the FSS, saving them in a protected (encrypted) form;
4. The *Data Processor* (DP), i.e., a medical personnel member (e.g., a doctor), who has to download the protected data from the FSS and has to be able to access and process them if he/she has the authorisation.

### 4.4. Medical-System-Controlled Access

The first solution is entirely managed by the medical system, which also regulates the access control to the protected data, and relies on an ElGamal-based PRE performed by the honest-but-curious cloud provider. Clearly, the use of a PRE technique implies an active role of the cloud provider, which has to perform the required operations to provide the correct execution of the mechanism. The primary motivation for using PRE is to relieve patients or the medical system of the burden of encrypting the data specifically for each member of the medical personnel to manage access to these data directly. In fact, PRE allows patients to encrypt their own data without the need to know its recipients, because the re-encryption process will make the ciphertext accessible to each one of them. Using conventional public key encryption schemes (e.g., RSA), instead, patients have to know the public key of the recipients of their medical data at the time they are encrypting the data. The PRE operation makes the ciphertext specific for each recipient, so nobody but the target recipient will be able to access the related content. Moreover, because of the properties of PRE, the Cloud Provider has no way to learn any information about the content of the ciphertexts during the re-encryption process.

With reference to the secure sharing architecture described in Section 4.3, the KMS is responsible for providing the public, re-encryption and private keys, respectively, to the patients, the Cloud Service Provider and the medical personnel. In particular, patients will use these public keys to encrypt data they want to share with medical personnel; the Cloud Service Provider will use the re-encryption keys to perform the PRE operations to make data accessible to specific members of the medical personnel, who will use their own private keys to access the data that patients share with them.

Being an ElGamal-based solution, all mathematical operations are meant to be executed in the modular arithmetic; in the following, we will omit the modulo only for the sake of readability.

#### 4.4.1. Cryptographic Primitives

This architecture makes use of an ElGamal-based cryptosystem consisting of the following seven algorithms.

GlobalSetup(λ)→GP The global setup algorithm takes in a security parameter λ and outputs global parameters GP for the system, consisting of the modulo *p* and a group generator $g\in Z_{p}^{+}$.
KeyGen()→(PubKey,PrivKey) The key generation algorithm outputs a public/private key pair, using the ElGamal key generation, with a random $s\in Z_{p}^{+}$ being the private key and $g^{s}$ being the public one.
Enc(PubKey,M)→C The encryption algorithm takes in a public key PubKey and a message *M* and, using the ElGamal encryption, outputs the ciphertext
  (1) $$C=(g^{r},M*g^{rs})$$
  where *r* is a blinding factor used in the ElGamal cryptosystem.
$PREKeyGen(g^{r})\to PREKey$ The PRE key generation algorithm takes in a blinding factor $g^{r}$ related to a specific ciphertext and computes a PRE key as
  (2) $$PREKey={\left(g^{r}\right)}^{x_{i}}=g^{rx_{i}}$$
  where $x_{i}\in Z_{p}^{+}$ is a random value.
$PREnc\left(PREKey,C\right)\to C^{\prime }$ The proxy re-encryption algorithm takes in a PRE key PREKey and a ciphertext *C* and computes
  (3) $$C_{\left(0\right)}^{\prime }=C_{\left(0\right)}$$
  (4) $$C_{\left(1\right)}^{\prime }=C_{\left(1\right)}*g^{rx_{i}}=M*g^{rs}*g^{rx_{i}}=M*g^{rs+rx_{i}}=M*g^{r(s+x_{i})}$$
  obtaining the new ciphertext
  (5) $$C^{\prime }=\left(C_{\left(0\right)}^{\prime },C_{\left(1\right)}^{\prime }\right)=\left(g^{r},M*g^{r(s+x_{i})}\right)$$
$DecKeyGen(g^{r})\to K$ The decryption key generation algorithm takes in a blinding factor $g^{r}$ related to a specific re-encrypted ciphertext and computes a decryption key as
  (6) $$K=g^{-r(s+x_{i})}$$
Dec(K,C)→M The decryption algorithm takes in a decryption key *K* and a ciphertext *C* and computes the plaintext as
  (7) M=C*K

#### 4.4.2. Data Protection and Storage

**Figure 7:**
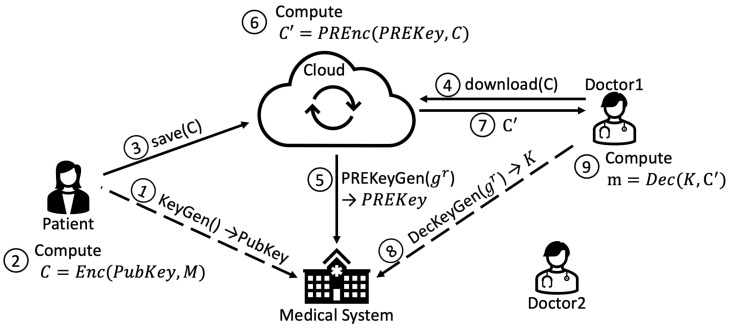
PRE-based data protection and access.

Files are uploaded to the FSS in an encrypted form using a *generic* public key. Steps 1–3 of Figure 7 detail the operations required to encrypt the data and store it in the cloud. The patient performs the following operations:

1. Asks the KMS for her public key;
2. Encrypts the plaintext;
3. Saves the ciphertext on the FSS.

#### 4.4.3. Data Access

Since data are encrypted with generic public keys, and we want to provide access individually, the ciphertexts have to be re-encrypted so that they can be accessed only by authorised users. To access a file, each member of the medical personnel needs a personal private key. When a member of the Medical Personnel, e.g., a doctor, wants to decrypt a ciphertext and access the content of the resulting plaintext, the operations required are the ones shown by steps 4–9 in Figure 7:

4. The doctor asks the FSS service to access a specific file.
5. The FSS service asks the KMS for a proxy re-encryption key for the specific file.
6. The FSS service applies a PRE operation and re-encrypts the file using the received proxy re-encryption key.
7. The FSS sends the new ciphertext to the doctor.
8. The doctor asks the KMS for his/her private key.
9. The doctor decrypts the ciphertext.

### 4.5. User-Controlled Access

Unlike the previous solution, the second one is user-controlled, as the access control is regulated by the patients thanks to the definition of access policies using CP-ABE. Due to CP-ABE, we can decouple the attributes of the receivers of medical data from their identities. Indeed, patients just have to know the characteristics of the doctors who are accessing their data. For example, if data about a cardiopathic patient must be accessible to the cardiologists of a specific hospital and their general practitioner (GP), the policy associated with the data could be (“CARDIOLOGIST” AND “ST. ANDREW HOSPITAL”) OR “GP”. This has the following advantages.

- Patients need to know only that their data can be inspected by the cardiologist, but neither their name nor their public keys are required.
- If the hospital recruits new cardiologists, it suffices to give them the attribute “CARDIOLOGIST” to allow them to access all the data that may be of interest to them.
- If a doctor loses the personal key, he/she can ask the medical system to reissue a new ABE secret key.

The main challenge is to properly handle key revocation, a problem that has nevertheless been tackled for a long time and has been addressed in several literature solutions [29,105]. This second architecture allows patients to define very specific access policies, providing flexible, fine-grained access control. Moreover, in contrast with the first solution, where a re-encryption operation is required to make the original ciphertext accessible to the recipient, with ABE, once encrypted, data are ready for sharing and access: there is no need for further operations on the protected data by any entity. Thus, there is no participation of the Cloud Provider, making it a passive entity whose only role is to share data obliviously among users.

With reference to the secure sharing architecture described in Section 4.3, the KMS is responsible for providing the ABE public keys and secret keys to the patients and medical personnel. In particular, patients will use these public keys to encrypt data that they want to share with medical personnel. Conversely, members of the medical personnel will use their own secret keys to access the data that patients share with them.

#### 4.5.1. Cryptographic Primitives

This architecture makes use of an CP-ABE cryptosystem presented in Section 2, and that is represented by the following five algorithms.

GlobalSetup(λ)→GP The global setup algorithm takes in the security parameter λ and outputs global parameters GP for the system.
KeyGen(GP)→(PubKey,MasterSecKey) The key generation algorithm setup algorithm with GP as input to produce its own master secret key and public key pair.
Enc(PubKey,M,Policy)→C The encryption algorithm takes in a public key PubKey, a message *M* and a Policy and it outputs a ciphertext *C*.
SecKeyGen(MastSecKey,AttrList)→SecKey The secret key generation algorithm takes in the master secret key MasterSecKey and an attribute list AttrList and outputs the secret key SecKey.
Dec(SecKey,C)→M The decryption algorithm takes in a secret key SecKey and a ciphertext *C*. If the decryption operation is successful, it generates the plaintext *M*; otherwise, ⊥ is returned.

#### 4.5.2. Data Protection and Storage

**Figure 8:**
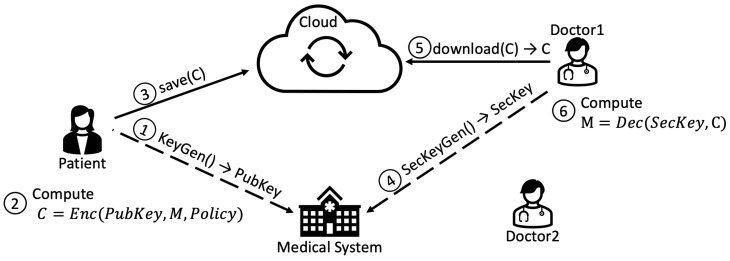
ABE-based data protection and access.

Files are uploaded to the FSS in an encrypted form, using ABE to grant fine-grained permission to a restricted group of people, for example, “CARDIOLOGIST”, “SURGEON”, or both. Since ABE is an asymmetric cryptosystem, to encrypt a new file, patients just need to use the ABE public key and specify the access policy. Steps 1–3 of Figure 8 show the operations required to encrypt the data and store it in the cloud. The patient performs the following operations:

1. Retrieves the ABE public key from the Medical System (KMS).
2. Encrypts the plaintext, defining and attaching a proper access policy to it.
3. Saves the ciphertext on the cloud (FSS).

#### 4.5.3. Data Access

To access a file, each member of the medical personnel needs an ABE secret key with a set of attributes that satisfies the specific access policy for whom the file was encrypted. When a member of the Medical Personnel, e.g., a doctor, wants to decrypt a ciphertext and access the content of the resulting plaintext, the operations required are the ones shown by steps 4–6 in Figure 8.

4. The doctor retrieves his/her own ABE secret key from the Medical System (KMS).
5. Downloads the desired file from the cloud (FSS).
6. Tries to decrypt the ciphertext using the secret key; then, if the set of attributes related to the secret key satisfies the access policy attached to the ciphertext, the decryption will be successful. Otherwise, the operation fails.

## 5. Performance Evaluation

As mentioned above, one of the drawbacks of the advanced cryptographic schemes proposed in this paper is the computational burden of the encryption and decryption operations of the asymmetric schemes. We, therefore, evaluated the computational costs of such operations in two scenarios: a Linux virtual machine (VM) in the cloud and a Raspberry Pi 3 B+ that could be used as a PoC for local data collection. The Linux VM employed uses the cores of a 3.0 GHz Intel Xeon Processor, while the Raspberry Pi 3 B+ uses a 1.2 GHz ARM Cortex-A53 processor. The programming language used to perform the tests is Python, and the chosen cryptographic libraries are *PyCryptodome* and *Charm*.

We evaluated the performance of three cryptographic schemes: CP-ABE, whose implementation is directly provided by Charm (hereafter referred to as *ABE*), and two ElGamal versions, a classical one based on modular arithmetics (hereafter referred to as *ElGamal*) and an optimised one based on elliptic curve cryptography (hereafter referred as *ECC ElGamal*), both developed from scratch using PyCryptodome primitives. Table 2 summarises all the details about the performance evaluation.

We measured the execution times of encryption/decryption operations applied to short messages (i.e., a 256-byte message). Long messages were not considered, since in this case hybrid encryption can be used; namely, the asymmetric scheme protects a relatively short symmetric key used to encrypt the message. Concerning the ABE encryption scheme, the policy used during the tests consisted of an *AND* operator between two string attributes. Using the *cpulimit* tool, the process CPU usage was limited during the execution of the tests. In particular, we evaluated four CPU usage percentages: 25%, 50%, 75% and 100%. It allowed us to simulate different load conditions of the system and to simulate devices with lower computational capabilities.

**Figure 9:**
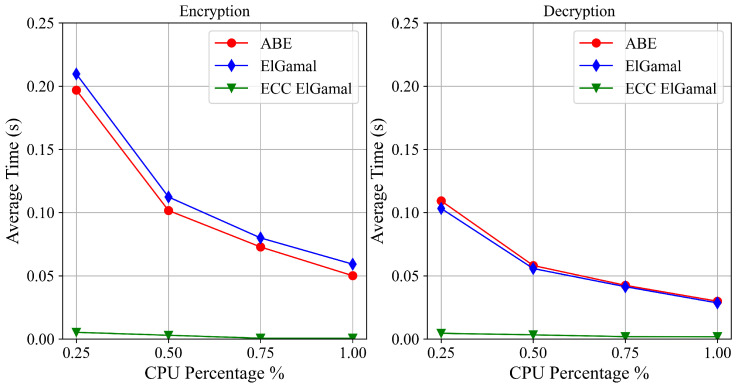
VM performance.

**Figure 10:**
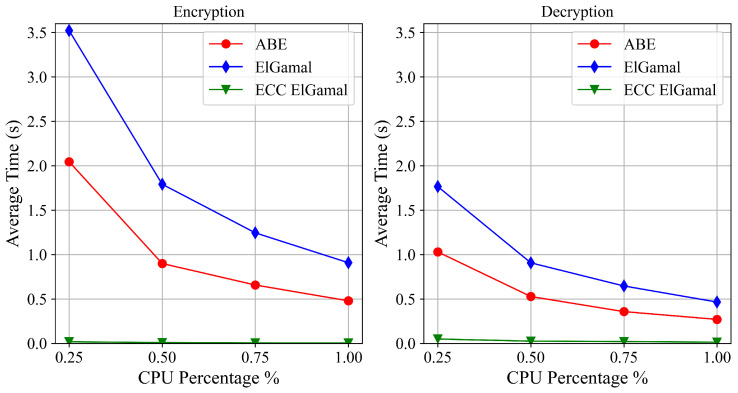
Raspberry Pi 3 B+ performance.

Figure 9 and Figure 10 show the average execution times of encryption and decryption operations of the three cryptographic schemes, respectively, on the VM and the Raspberry Pi 3 B+. At first glance, it is evident that ECC ElGamal outperforms the other two schemes. However, looking at the times reported in Figure 9, all three cryptographic schemes are viable solutions for data protection in a scenario with devices similar to the used VM. The same is not valid for the Raspberry Pi 3 B+. In fact, in the case of a high number of operations, ECC ElGamal is the only viable solution; however, the other two schemes remain feasible approaches if the number of operations is quite low.

## 6. Conclusions and Future Works

In this paper, the security of the data collected by NFC and RFID medical sensors and then shared with doctors via a cloud service is addressed through privacy-aware system architectures for the first time. For the first part of the telemonitoring system, viz., the wireless communications between sensing tags and readers, the most threatening attacks are identified through the scientific literature, and a set of known countermeasures is proposed. The conceived defence of tag-reader communications hampers skimming, man-in-the-middle, eavesdropping, power analysis, and timing attacks. For the messages from the reader to the doctor, two secure sharing architectures that can exploit even commercial cloud services are instead envisaged. The first architecture is controlled by the medical system and exploits HE and PRE; in the second architecture, the data access is instead managed directly by the patients, thanks to CP-ABE. In both cases, the cloud provider cannot access the data because of the encryption of the messages containing the sensitive data. Depending on the actual needs of the patient and the target healthcare application, both solutions can be exploited to guarantee security and privacy while using NFC or RFID sensors to deploy, for instance, domestic points of care.

Before concluding the paper, it is relevant to outline some possible future works on secure healthcare systems using NFC and UHF RFID. The encryption capability of the latest generation of tags’ chips and their security should be investigated in-depth [103,104]. The system architectures described here should be implemented and tested in real scenarios involving healthcare systems and chronically-ill patients in order to assess the practical effectiveness beyond the theoretical soundness. Lastly, more advanced system architectures could be designed to exploit even implanted NFC and RFID sensors.

## Figures and Tables

**Table 1:** Literature search regarding security and privacy in healthcare through NFC/RFID or data sharing architectures. The symbol “*” denotes that all the results including the given substring are included.

Search Terms	Initial Search	1st Screening	2nd Screening	Merge Searches
“RFID” AND “health * ”AND “security”	990	882	605	-
“RFID” AND “health *” AND “privacy”	539	480	325	-
“NFC” AND “health *” AND “security”	154	144	98	-
“NFC” AND “health *” AND “privacy”	44	38	28	-
**Total−“RFID”-“NFC”**	1727	1544	1056	764
“health *” AND “cloud” AND “security”	6803	6232	4276	-
“health *” AND “cloud” AND “privacy”	4371	4024	2725	-
**Total−“Data Sharing”**	11,174	10,256	7001	4936

**Table 2:** Performance evaluation specifications.

**Scenarios**	**Used Device**	**CPU**
	Virtual Machine	Intel Xeon Processor
		3.0 GHz, 64 bit
	Raspberry Pi 3 B+	ARM Cortex-A53
		1.4 GHz, 64 bit
**Measured performance**	Execution Time	
**Programming language**	Python	
**Cryptographic libraries**	1. PyCryptodome, 2. Charm	
**Cryptographic schemes**	1. ABE. 2. ElGamal, 3. ECC ElGamal	

## Data Availability

The dataset of the literature review is available from the authors.
